# Monocyte subpopulations display disease-specific miRNA signatures depending on the subform of Spondyloarthropathy

**DOI:** 10.3389/fimmu.2023.1124894

**Published:** 2023-04-17

**Authors:** Małgorzata Stec, Marcin Czepiel, Marzena Lenart, Agata Piestrzyńska-Kajtoch, Jacek Plewka, Agnieszka Bieniek, Kazimierz Węglarczyk, Rafał Szatanek, Magdalena Rutkowska-Zapała, Zofia Guła, Anna Kluczewska, Jarosław Baran, Mariusz Korkosz, Maciej Siedlar

**Affiliations:** ^1^ Department of Clinical Immunology, Institute of Pediatrics, Jagiellonian University Medical College, Krakow, Poland; ^2^ Department of Animal Molecular Biology, National Research Institute of Animal Production, Balice, Poland; ^3^ Department of Chemistry, Jagiellonian University, Krakow, Poland; ^4^ Department of Rheumatology and Immunology, Jagiellonian University Medical College, Krakow, Poland

**Keywords:** Spondyloarthropathy, monocytes, miRNA, monocyte subpopulations, rheumatic disorders

## Abstract

Spondyloarthropathies (SpA) are a family of rheumatic disorders that could be divided into axial (axSpA) and peripheral (perSpA) sub-forms depending on the disease clinical presentation. The chronic inflammation is believed to be driven by innate immune cells such as monocytes, rather than self-reactive cells of adaptive immune system. The aim of the study was to investigate the micro-RNA (miRNA) profiles in monocyte subpopulations (classical, intermediate and non-classical subpopulations) acquired from SpA patients or healthy individuals in search for prospective disease specific and/or disease subtype differentiating miRNA markers. Several SpA-specific and axSpA/perSpA differentiating miRNAs have been identified that appear to be characteristic for specific monocyte subpopulation. For classical monocytes, upregulation of miR-567 and miR-943 was found to be SpA-specific, whereas downregulation of miR-1262 could serve as axSpA-differentiating, and the expression pattern of miR-23a, miR-34c, mi-591 and miR-630 as perSpA-differentiating markers. For intermediate monocytes, expression levels of miR-103, miR-125b, miR-140, miR-374, miR-376c and miR-1249 could be used to distinguish SpA patients from healthy donors, whereas the expression pattern of miR-155 was identified as characteristic for perSpA. For non-classical monocytes, differential expression of miR-195 was recognized as general SpA indicator, while upregulation of miR-454 and miR-487b could serve as axSpA-differentiating, and miR-1291 as perSpA-differentiating markers. Our data indicate for the first time that in different SpA subtypes, monocyte subpopulations bear disease-specific miRNA signatures that could be relevant for SpA diagnosis/differentiation process and may help to understand SpA etiopathology in the context of already known functions of monocyte subpopulations.

## Introduction

Spondyloarthropathies (SpA) are a family of rheumatic disorders characterized by chronic inflammation within spine, peripheral joints and entheses with resultant unfavourable remodelling of the skeleton. Phenotypically, SpA could be divided into axial sub-form (axSpA) involving mainly joints of the spine, and peripheral SpA (perSpA), affecting peripheral skeleton with common clinical manifestations, including arthritis, enthesitis and dactylitis. Emerging data from immunopathology studies and clinical trials indicate that axial and peripheral SpA might be driven by different mechanisms and respond differently to treatment (1). In line with that, genetic, histopathological, and clinical evidences indicate that despite common downstream pathways, mediated e.g. by macrophage-derived TNF, inflammation in SpA is driven and maintained by different cellular and molecular mediators (2, 3). Moreover, it has been recently proposed that SpA is an autoinflammatory disease driven by innate immune cells i.a. monocytes, rather than a genuine autoimmune disease triggered by self-reactive T and/or B lymphocytes (4), although certain phenomena of autoimmunity in pathogenesis of ankylosing spondylitis (AS), which is the model entity of axSpA, are also considered (5). Thus, different pathophysiology and clinical course of axial and peripheral SpA might be affected by changes in the count and/or percentage of populations of circulating mononuclear cells, their products and/or proinflammatory activity. Nevertheless, in the course of SpA, the pathophysiological role of specific monocyte subpopulations (i.e. classical CD14++CD16-, intermediate CD14++CD16+ and non-classical CD14+CD16++ monocytes) as a source of pro- and anti-inflammatory mediators as well as their impact on disease severity has not been fully elucidated. On the other hand, in other rheumatic diseases, e.g. rheumatoid arthritis (RA), increased monocyte count (especially CD14+CD16++ subpopulation) correlates with clinical manifestations and elevated parameters of inflammation localized in peri-articular tissue (6–8). Moreover, dendritic cells originated from migrating monocytes seem to play a significant role in pathogenesis of rheumatic inflammatory processes and participate in osteogenesis and inflammation-mediated destruction of bone tissue (9). In line, monocytes seem to favour maintenance of inflammation in peri-articular tissues in patients with AS (10), and the classical CD14++CD16- monocyte subpopulation is believed to be the source of osteoclasts in patients with RA (11, 12).

MicroRNAs (miRNAs) are small endogenous, non-coding RNAs that regulate gene expression at post-transcriptional level. They are involved in a range of physiological and pathological processes associated with immune regulation and development of autoimmunity. Dysregulated expression of miRNAs has been described in numerous rheumatic disorders including Spondyloarthropathies (13, 14). Nevertheless, here we show, for the first time, differential expression of miRNAs in monocyte subpopulations from SpA patients suffering from either peripheral or axial disease. Considering the critical role of miRNAs in the regulation of innate immune system together with their apparent contribution to pathological processes observed in two subforms of SpA, the obtained results could help complete the picture of SpA pathogenesis in the context of already known monocyte functions.

## Materials and methods

### Patients

Forty-six patients with SpA,27 axial SpA according to the Assessment of SpondyloArthritis International Society (ASAS) classification criteria for axial SpA 2009 and 19 peripheral SpA according to ASAS classification criteria for peripheral SpA 2011 (15, 16) and 20 healthy age- and sex-matched subjects were enrolled in the study (HC – healthy controls). Patients were under 45 years, naive to synthetic, synthetic-targeted, or biologic Disease Modifying Anti-Rheumatic Drugs (DMARDs) and without administration of systemic glucocorticosteroids. Patients provided a signed informed consent, and the study protocol was approved by the local Bioethics Committee (KBET/252/B/2012).

**Table 1** presents characteristics of patients. Briefly, median age (years, IQR) of axSpA patients was (29.7-39.7) and perSpA patients was (31-38.5). Median disease duration (years, IQR) was (5-10.7) for axSpA and (2-9.5) for perSpA patients. 81% of axSpA and 37% of perSpA patients were HLA-B27 positive. Twenty-two (81%) axSpA patients fulfilled mNY criteria for AS.

**Table 1:** Demographic and clinical characteristics of patient groups.

	axial SpA (n= 27)	peripheral SpA (n= 19)	p-value
Age (years), median (IQR)	33.5 (26.0-38.8)	35.5 (31.5 – 38.5)	NS
Males, n (%)	16 (59)	11 (58)	
HLA B27 positive n (%)	22 (81%)	7 (37%)	0.03
Disease duration (years), median (IQR)	4 (3-8)	2 (0.5-6)	NS
ESR mm/h, median (IQR)	22.5 (15.5-30.8)	25 (17-42)	NS
CRP mg/l, median (IQR)	8.8 (2.4-12.9)	7.4 (2.6 – 12.6)	NS
BASDAI median (IQR)	2.05 (0.9- 4.3)	3.95 (2.4-6.4)	0,03
ASDAS (CRP) median (IQR)	2.14 (1.6-2.9)	2.65 (2.1-3.5)	NS
IBP (inflammatory back pain), n (%)	23 (85)	7 (40)	<0,001
Number of swollen joints (out of 66), median (IQR) Number of painful joints (out of 68), median (IQR)	0 0	2 (1-3.8) 2 (1-4.5)	<0.001 <0.001
DAS28 (ESR) median (IQR)	NA	4.0 (3.0-4.4)	NA
Enthesitis n (%)	7 (26)	13 (68)	0,045
Dactylitis n (%)	1 (4)	17 (89)	<0.001
Diagnosis, n (%) nr axSpA AS PsA per SpA	5 (19) 22 (81) 0 0	0 0 10 (53) 9 (47)	

BASDAI, Bath Ankylosing Spondylitis Disease Activity Index; ASDAS, Ankylosing Spondylitis Disease Activity Score; DAS28, Disease Activity Score (ESR) 28; IBP, inflammatory back pain; CRP, C-reactive protein; ESR, erythrocyte sedimentation rate; ax, axial, nr, non radiographic; per, peripheral, SpA, spondyloarthritis; AS, ankylosing spondylitis; PsA, psoriatic arthritis. NS, not significant.

### Isolation of monocytes and their subsets

Monocyte subpopulations were isolated from peripheral blood mononuclear cells (PBMC) obtained from SpA patients or healthy donors. PBMC were isolated from EDTA-treated whole peripheral blood by the standard Pancoll human (Panbiotech, Aidenbach, Germany; P04-60500) density gradient centrifugation. PBMC were washed in PBS (Sigma-Aldrich, Saint Louis, USA) and then monocyte subsets (classical - CD14^++^CD16^-^, intermediate - CD14^++^CD16^+^ and non-classical - CD14^+^CD16^++^) were isolated using flow cytometry cell sorting. The following monoclonal antibodies (mAbs) were used to stain monocytes: anti-CD14-FITC (clone MφP9, BD Bioscience), anti-CD16-PE (clone 3G8, BD Bioscience) and anti –HLA -DR -PerCP (clone L243, BD Buoscience), in 1:25 dilution (v/v) and gated as previously described (17, 18). The monocytes were then incubated for 30 min at 4°C, followed by sorting using the FACSAria II cell sorter (BD Biosciences, San Jose, CA, USA). Sorter was equipped with 488 nm and 561 nm lasers for excitation of FITC, PE and PerCP. The following band-pass filters were used for the measurement of fluorescence: 530/30 for FITC, 695/40 for PerCP (laser 488 nm) and 582/42 for PE (laser 561 nm). After isolation, the cells were washed in PBS, centrifuged for 10 min at 350 x g and kept frozen at -80°C until RNA isolation. The absolute numbers of FACS-sorted monocyte subpopulations were previously provided (19).

### QuantStudio OpenArray MicroRNA Expression

miRNA expression was analyzed on QuantStudio 12KFlex Real-time PCR System with OpenArray (OA) block (Applied Biosystems by ThermoFisher Scientific, Waltham, MA, USA) with TaqMan OpenArray Human miRNA Panel (P/N: 4470187) according to OpenArray MicroRNA Expression Workflow and reagents by ThermoFisher Scientific (Waltham, MA, USA) (**Supplementary figure 1**). Each OA plate enables the quantification of miRNA expression (panel A and B) in 3 samples and four OA plates can be run together what allows simultaneous analysis of 12 samples. Both panels together contain 754 human miRNA sequences from the Sanger miRBase v14, including target negative control (ath-miR159a) and target controls (U6 rRNA, RNU48, RNU44). Full list of miRNAs examined in this study is provided in **Supplementary File 3**.

RNAs were isolated using miRVana microRNA isolation kit (Thermo Fisher Scientific; AM1560) and transcripted into cDNA using Megaplex™ RT Primers and TaqMan^®^ MicroRNA Reverse Transcription Kit (Thermo Fisher Scientific; 4366597). Next, the cDNA was pre‐amplified to increase its quantity before performing the OpenArray qPCR. Preamplification products were prepared in two separate reactions for Megaplex™ PreAmp Primers Pool A and Pool B, corresponding to the Megaplex™ RT Primers Pool used previously for reverse transcription. For each pre-amplification we used 2,5 µl cDNA and 22,5 μL of PreAmp Reaction Mix, containing Megaplex™ PreAmp Primers Pool A or Pool B (2,5 µl), 2x TaqMan^®^ PreAmp Master Mix (12,5 µl) and nuclease-free water (all reagents by ThermoFisher Scientific, Waltham, MA, USA). Pre-amplification reaction tubes with reagents were incubated on ice for 5 minutes before performing RUN on SimplyAmp Thermal Cycler (Applied Biosystems by ThermoFisher Scientific, Waltham, MA, USA). The following thermal conditions were used to run pre-amplification: 95°C for 10 minutes, 55°C for 2 minutes, 72°C for 2 minutes, 12 cycles including 95°C for 15 seconds and 60°C for 4 minutes, hold step with 99,9°C for 10 minutes and final hold step 4°C. Negative control was used for each preamplification. Every single preamplification product was diluted 1:20 with nuclease-free water and used for the next step within 12 hours. For each sample, qPCR reaction was prepared by mixing 22,5 µl 2x TaqMan^®^ OpenArray^®^ Real‐Time PCR Master Mix and 22,5 µl pre-amplification product on 96-well plate. Next, the qPCR reaction mixes with samples (5 µl) were transferred by pipetting to 384-well plate according to protocol created in OpenArray Sample Tracker Software (ThermoFisher Scientific, Waltham, MA, USA) and loaded to OpenArray plates by OpenArray™ AccuFill™ System (ThermoFisher Scientific, Waltham, MA, USA). OpenArray cases were sealed according to guidelines within 90 seconds. Ready OpenArray Plates were put into the QuantStudio 12K Flex Real-time PCR System with OpenArray block and processed.

After processing OpenArray miRNA Expression run, the data analysis was performed. Quality control images (QC Images) were exported and investigated to find potential loading errors. The result files created by QuantStudio Software v1.2.3 were uploaded to ThermoFisher Cloud and analyzed with Relative Quantification qPCR Application. miRNA Panel A and B were analyzed in separate analysis groups.

### Statistical analysis

The expression of each miRNA type was calculated using 2^−ΔΔCT^ method and snRNa U6 was used as a control to all miRNA analysis. The results were shown using volcano plot (p-value vs. fold change) with following settings: fold change boundry 2,0, p-value boundry 0,05. Median values for each group were compared using Kruskal-Wallis test. Data analysis was performed in Mathematica 12 software (Wolfram Research, Inc., Mathematica, Version 12.0.0, Champaign, IL, USA) and GraphPad Prism version 9 (GraphPad Software Inc., San Diego, CA). The principal Component Analysis was performed in SPSS Statistisc software (version 29.0.0.0 (241)).

## Results

SpA patients fulfilling the study inclusion criteria were enrolled. Patients were under 45 years, naive to synthetic, synthetic-targeted or biologic Disease Modifying Anti-Rheumatic Drugs (DMARDs) and without administration of systemic glucocorticosteroids. Detailed patients’ characteristics is presented in **Table 1** (Methods section). Further, monocytes from patients’ peripheral blood were isolated and divided into separate subpopulations using FACS, which was followed by RNA isolation and gene expression analysis with quantitative PCR miRNA array.

**Figure 1:**
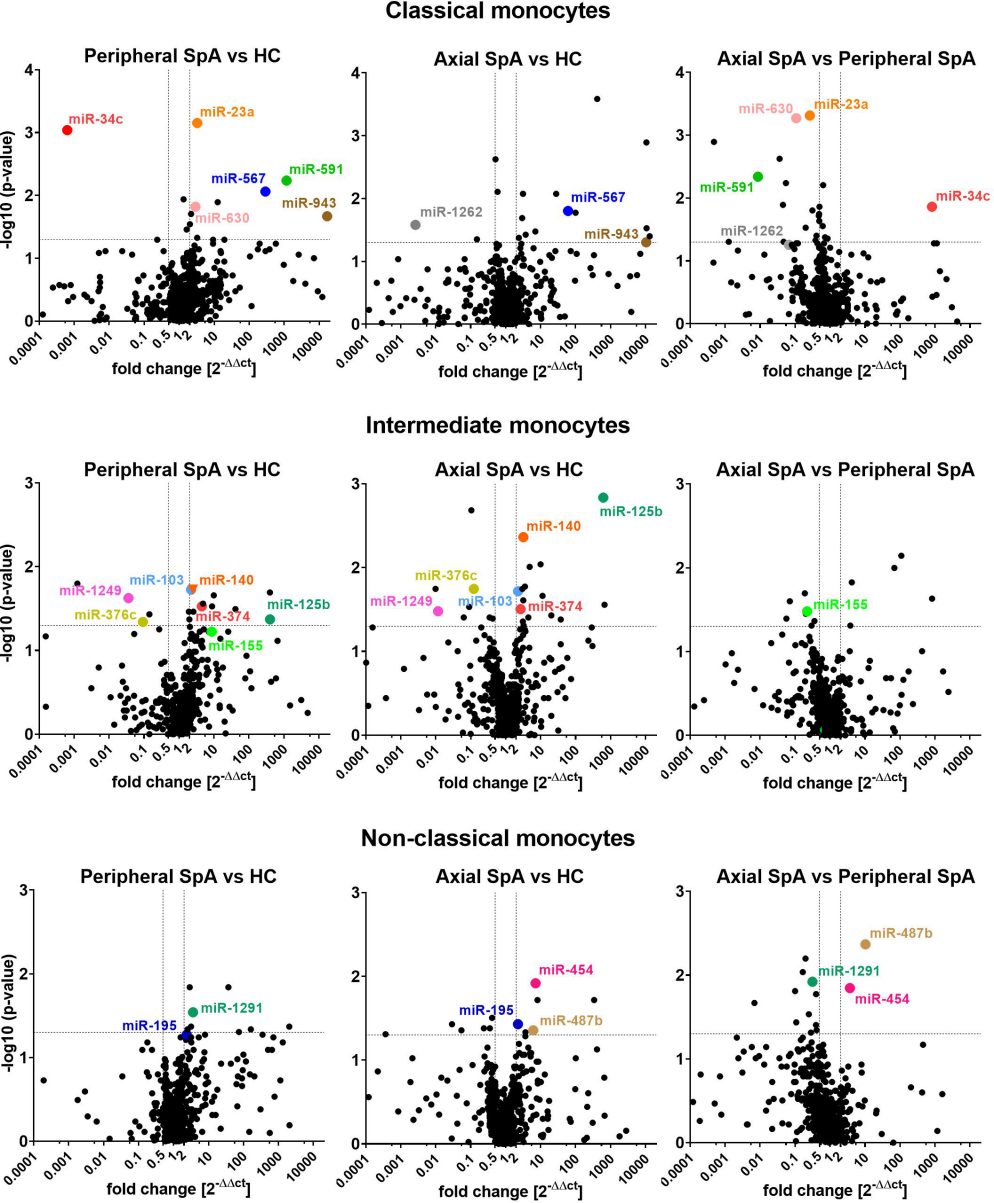
Volcano plots comparing average miRNAs expression levels in classical (top), intermediate (middle) and non-classical monocytes (bottom) obtained from perSpA and axSpA patients and healthy donors. Differentially expressed miRNAs meeting inclusion criteria (see results section) have been highlighted.

In the analyses we focused on statistically different miRNAs expression between the SpA subtypes and healthy subjects (3 groups: axSpA, perSpA, HC) within monocyte subpopulations, that were upregulated at least by 2 fold or downregulated at least by 0.5 fold. We also selected certain miRNAs that complied with above criteria in 2 out of 3 studied groups (e.g. upregulation of miR-567 in axSpA vs. HC and perSpA vs. HC, but not in axSpA vs. perSpA groups). Such an approach allowed for identification of differentially expressed miRNAs, whose expression (down- or upregulation) in monocyte subpopulations would be typical for SpA (independent of the disease variant), or could be characteristic for the specific disease subtype (axSpA vs. perSpA and HC, or perSpA vs. axSpA and HC). The analysis results are shown as volcano plots in **Figure 1**. The highlighted miRNAs fulfilled the analysis criteria.

Several SpA-specific and axSpA/perSpA differentiating miRNAs have been identified that appeared to be characteristic for monocyte subpopulations. For classical monocytes, upregulation of miR-567 and miR-943 was found to be SpA-specific, whereas downregulation of miR-1262 could serve as axSpA-differentiating, and expression pattern of miR-23a, miR-34c, mi-591 and miR-630 as perSpA-differentiating markers. For intermediate monocytes, expression levels of miR-103, miR-125b, miR-140, miR-374, miR-376c and miR-1249 could be used to distinguish SpA patients from healthy donors, whereas expression pattern of miR-155 was identified as characteristic for perSpA. For non-classical monocytes, differential expression of miR-195 was recognized as general SpA indicator, while upregulation of miR-454 and miR-487b could serve as axSpA-differentiating, and miR-1291 as perSpA-differentiating markers. Furthermore, the associations between significantly differentially expressed miRNAs themselves has been investigated (**Supplementary File 4**) as well as Principal Component Analysis (PCA) has been performed in search for combined miRNA signatures that could potentially differentiate SpA categories from healthy controls. PCA revealed that expression patterns of combined miR-23a and miR-630 in classical monocyte subset could serve as a good discriminator between perSpA vs. axSpA and HC. Expression pattern of miR-1249 in intermediate monocyte subset could well discriminate between HC vs. axSpA and perSpA. Combined expression patterns of miR-195 and miR-1291 in non-classical monocyte subset could serve as a good discriminator between axSpA vs. perSpA and HC. On the other hand, differential expression of miR-487b could be used to discriminate between axSpA vs. HC. The results form PCA correspond well with the data presented in **Figure 1**. Interestingly, when all differentially expressed miRNAs were considered for PCA, the group or miRNAs (namely: miR-148b, miR-324-5p, miR-130b, miR-17, miR-30b, miR-1255, miR-302c, miR-26a-3p) has been identified that combined expression pattern in classical monocytes could discriminate between perSpA vs. axSpA and HC (PCA details available in **Supplementary File 5**).

Additionally, we examined whether expression of above-identified miRNAs correlates with patients’ data and characteristic SpA clinical features. The identified significant correlations are summarized in **Table 2**.

**Table 2:** Spearman correlations between miRNA expression and SpA patients’ clinical data.

Monocyte subpopulation	SpA subform	miRNA	SpA parameter	Spearman r	95% CI	p -value
*Classical*	Axial SpA	miR-567	ESR CRP ASDAS	0.50 0.66 0.46	0.056 to 0.776 0.330 to 0.853 0.028 to 0.741	0.0257 0.0007 0.0332
		miR-27b	ASDAS BASDAI Enthesitis DXA_FN	0.46 0.57 0.53 -0.71	0.000 to 0.765 0.155 to 0.813 0.100 to 0.793 n/a	0.0446 0.0089 0.0163 0.0270
	Peripheral SpA	miR-302c	ESR RTG_SIJ ASDAS	0.58 -0.59 -0.57	0.097 to 0.839 -0.848 to -0.032 -0.852 to -0.100	0.0215 0.0218 0.0382
*Intermediate*	Peripheral SpA	miR-155	Disease duration DAS28	-0.64 0.58	-0.866 to -0.193 0.058 to 0.855	0.0092 0.0318
*Non-classical*	Axial SpA	miR-195	DXA_NF DXA_LS Disease duration Enthesitis	0.73 0.85 -0.48 -0.53	n/a n/a -0.765 to -0.031 -0.793 to -0.100	0.0204 0.0030 0.0332 0.0163
	Peripheral SpA	miR-487b	CRP ASDAS Enthesitis	-0.77 -0.81 -0.62	-0.912 to -0.484 -0.942 to -0.448 -0.864 to -0.145	<0.0001 0.0014 0.0193

ASDAS, Ankylosing Spondylitis Disease Activity Score; BASDAI, Bath Ankylosing Spondylitis Disease Activity Index; CRP, C-reactive protein level; DAS28, Disease Activity Score (ESR) 28; DXA_FN, Bone density – femoral neck; DXA_LS, Bone density – lumbar spine; Enthesitis, presence of Enthesitis; ESR, erythrocyte sedimentation rate; RTG_SIJ, degree of sacroilitis on x-ray images; n/a, not applicable.

We found that in classical monocytes expression of miR-567 positively correlates with such parameters as ESR (r=0.5, p=0.0257), CRP (r=0.66, p=0.0007) and ASDAS (r=0.46, p=0.0332) in axSpA patients. Further, for axSpA we observed strong negative correlations between expression of miR-31 and disease duration (r=-0.59, p=0.0044), as well as for expression of miR-30b vs. CRP (r=-0.64, p=0.0073) and degree of sacroilitis on x-ray images (r=-0.73, p=0.0011), according to modified New York criteria (cyt). On the other hand, expression of miR-27b cluster positively correlated with ASDAS (r=0.46, p=0.0446), BASDAI (r=0.57, p=0.0089) and the presence of enthesitis (r=0.53, p=0.016), but negatively with the bone density of the femoral neck (r=-0.71, p=0.027). For peripheral SpA, expression of single miRNA – miR-302c – positively correlated with ESR (r=0.58, p=0.021) and negatively with degree of sacroilitis on x-ray images (r=-0.59, p=0.022) and ASDAS (r=-0.57, p=0.038). Similarly, for intermediate monocytes, an interesting correlation between miR-155 expression levels and disease duration (r=-0.64, p=0.0092), as well as DAS28 score (r=0.58, p=0.0318) was detected. Finally, in non-classical monocytes isolated from axSpA patients, expression of miR-195 correlated positively with bone density of lumbar spine (r=0.85, p=0.0030) and of the femoral neck (r=0.73, p=0.0204) and negatively with disease duration (r=-0.48, p=0.0332) and the presence of enthesitis (r=-0.53, p=0.0163). In perSpA patients, there were significant negative correlations identified between CRP (r=-0.77, p<0.0001), ASDAS (r=-0.81, p=0.0014) and the presence of enthesitis (r=-0.62, p=0.0193) and the expression levels of miR-487b.

## Discussion

Dysregulation of miRNA expression is a common phenomenon that accompany numerous human diseases including, among others, immune deficiencies, neurodegenerative disorders and cancer (20–22). Similarly, in the course of chronic rheumatic inflammatory disorders such as SpA, miRNA profiles may vary indicating their influence in the underlying pathological processes (14). Here, we showed that SpA is not only a heterogeneous disease but also, we unveiled that immune processes associated with the body’s response towards the ongoing SpA pathology might be related to the alteration of specific miRNA landscapes. We demonstrated that, in SpA, different monocyte subpopulations harbor distinct miRNA expression profile characteristic for the specific subform of the disease. Several of the identified differentially expressed miRNAs have been already linked to rheumatic disorders. Interestingly, miR-34c, whose strong repression in perSpA classical monocytes was observed, has been found to be upregulated in Rheumatoid Arthritis (RA) PBMCs (23). miR-34c has been long recognized as a strong osteogenic inhibitor (24, 25), and hence its role in perSpA is likely to be related to the inflammation-driven bone structure remodeling. Similarly, the involvement miR-23a cluster - upregulated in perSpA classical monocytes – in such processes as regulation of bone formation/destruction has been described (26, 27). Additionally, miR-23a was implicated in IL-17 driven inflammation demonstrating an inhibitory effects on expression of IL-17-mediated proinflammatory mediators (28). In immune system, miR-23a cluster plays a decisive role in promoting myelopoiesis over lymphopoiesis (29) and has been shown to have a profound impact on the function of myeloid cells, including M-CSF-induced differentiation of monocytes to macrophages (30) as well as myeloid cells activation (31). Intriguingly, the member of the miR-23a paralog cluster – miR-27b – has been identified in our study to be strongly upregulated in axSpA classical monocytes compared to the healthy counterparts. This miRNA has been i.a. linked with such rheumatic diseases-relevant processes as enhancing osteogenesis of human mesenchymal stem cells (32), inhibiting IL-17-induced monocyte chemoattractant protein-1 (MCP1) expression (33) or suppressing the NF-κB signaling pathway in osteoarthritic chondrocytes (34). Notably, miR-27b expression levels correlated with number of disease parameters (**Table 2**) indicating an important role of this miRNA in the disease activity, enthesitis and bone remodeling processes occurring in axSpA. Scarce SpA-related data is available on other miRNAs (i.e. miR-302c, miR-567, miR-591, miR-630, miR-943 and miR-1262) found to be significantly differentially expressed in classical monocytes among study groups indicating the yet unexplored role of these miRNAs in rheumatology research, possibly as novel SpA biomarkers. In addition, as 80-90% of classical monocytes migrate from peripheral blood to the target tissues ca. 1 day after being released from the bone marrow (35), their harboured miRNA signatures may exert significant impact on the cytokine milieu of the local inflamed tissues, i.e. synovitis, osteitis and enthesitis.

Vast majority of differentially expressed miRNAs in intermediate monocytes occurred to be specific for SpA in general. In fact, upregulation of only single miRNA, namely miR-155, has been found to be characteristic for perSpA. Furthermore, the expression levels of miR-155 correlated with disease duration (negatively) and DAS28 (positively) in perSpA. Dysregulation of miR-155 has been already associated with rheumatic disorders including RA (36) and spondyloarthritis (37). For instance, miR-155 is highly expressed in synovial fluid-derived monocytes/macrophages compared with the peripheral blood counterparts from patients with RA. Incubation of peripheral blood CD14+ cells with RA synovial fluid stimulated the expression of miR-155 and release of TNF-alpha; while the cytokine production was downregulated by transfection of miR-155 inhibitor (38). Moreover, miR-155 is commonly believed as highly pro-inflammatory miRNA contributing to impaired Treg function (39), augmentation of Th17 response (38) and support of M1 macrophage polarization (40). Thus, our finding that miR-155 expression levels are at its highest at early stages of perSpA (inflammatory disease phase) and then gradually decrease with the course of the disease, seems to be consistent with the role of miR-155 as a critical driver of inflammatory mechanisms. Other differentially expressed intermediate monocyte miRNA – miR-103, miR-125b, miR-140 (all upregulated in SpA patients in comparison to HC) – have been implicated to play diverse functions in development of several rheumatic disorders including osteoarthritis [miR-103 (41), miR-125b (42), miR-140 (43)], RA [miR-103 (44), miR-125b (45), miR-140 (46)] and juvenile idiopathic arthritis [miR-125b (47)]. Certain common features of the rheumatic diseases would suggest that the abovementioned miRNAs may as well be important in the pathogenesis of SpA. Interestingly, in a mouse model of axSpA, miR-103 levels were found elevated in animals subjected to reduced mechanical loads. Moreover, the increase of miR-103 expression led to upregulation of the potent osteogenesis inhibitor - Dkk-1 - and hence, reduced new bone formation/bone density in SpA mice (48). Whether similar, miR-103-driven mechanism operates also in human axSpA, remains to be verified.

In non-classical monocytes, only four differentially expressed miRNAs meeting the inclusion criteria have been identified, namely miR-195, miR-454, miR-487b and miR-1291. Little is known about miR-454 and miR-1291 in context of rheumatic disorders. On the other hand, miR-195 (upregulated in SpA vs. control monocytes) and miR-487b (upregulated in axSpA vs. perSpA and control monocytes) could be linked to SpA pathophysiology primarily through their influence on osteogenesis (miR-195) and inflammation (miR-487b). Several studies showed the pro-osteogenic function of miR-195 (49, 50), which corresponds well with the positive correlation of miR-195 levels and bone density observed in examined axSpA patients. Furthermore, the opposite relation of miR-195 expression with the disease duration might indicate its importance in mechanisms leading to the loss of bone density observed at later disease stages. miR-487b, in contrary, is considered a potent inhibitor of inflammation processes (51, 52). Consistently, a recent study revealed that miR-487b-laden extracellular vesicles possess a potent anti-inflammatory activity, likely through suppression of MAPK signaling pathway (53). Such a function might well explain the negative correlation of miR-487b levels and inflammation-related disease parameters identified in studied perSpA cohort. Furthermore, miR-487b has been found to play an important role in bone metabolism. Thus, impairment of osteoblastogenesis by interference with Notch-1 signaling might indicate its engagement in dysregulation of bone turnover processes observed in SpA (54).

In conclusion, the results of the study clearly demonstrate the dysregulation of miRNA signatures in SpA monocytes compared to their healthy counterparts. More, we showed for the first time, that miRNA profiles of monocyte subpopulations differ significantly depending on the predominant disease pathology. These results may therefore be of significant diagnostic value, especially at initial disease stages, when the definite distinction of SpA subvariant is problematic due to the lack of yet extensive remodeling of the bone tissue. Noticeably, the pathomechanisms governing the peripheral and the axial form of SpA seem to be very different, hence the proper, early recognition of the disease subvariant could be critical in terms of disease prognosis and choosing appropriate treatment strategy and expected response to the applied therapy, eventually resulting in either success or failure of the treatment.

## Data availability statement

The original contributions presented in the study are publicly available. This data can be found here: https://www.ncbi.nlm.nih.gov/geo/query/acc.cgi?acc=GSE223717.

## Ethics statement

The studies involving human participants were reviewed and approved by Bioethics Committee of Jagiellonian University. The patients/participants provided their written informed consent to participate in this study.

## Author contributions

MSi – designed and supervised the study MSt, MC, MSi – wrote the manuscript ML, JP, AP-K, ZG, JB, MK, MSi – corrected the manuscript MSt, MC, ML, AP-K, AB, KW, RS, MR-Z, AK – performed the experimental work MSt, MC, ML, JP – analyzed the data MK, ZG – recruited SpA patients. All authors contributed to the article and approved the submitted version.

## Supplementary material

Supplementary Figure 1 FACS gating strategy for isolation of classical, intermediate and non-classical monocyte subsets. P1 – gate containing monocytes and NK cells; P2 – gate containing monocytes; P3,P4, P5 – gates containing non-classical, intermediate and classical monocytes respectively.

Supplementary Figure 2 OpenArray MicroRNA Expression Workflow presenting methodological aspects of miRNA expression analysis in human monocyte subpopulations.

Supplementary File 3 Full list of miRNAs examined in this study.

Supplementary File 4 Spreadsheets presenting associations between differentially expressed miRNAs themselves in classical, intermediate and non-classical monocyte.

Supplementary File 5 Detailed description of principal component analysis of differentailly expresssed miRNAs.
