# Supplementary figures and images for: Monocyte subpopulations display disease-specific miRNA signatures depending on the subform of Spondyloarthropathy
Source: Front Immunol. 2023 Apr 17;14:1124894. doi: 10.3389/fimmu.2023.1124894 (PMC10149963; doi:10.3389/fimmu.2023.1124894)

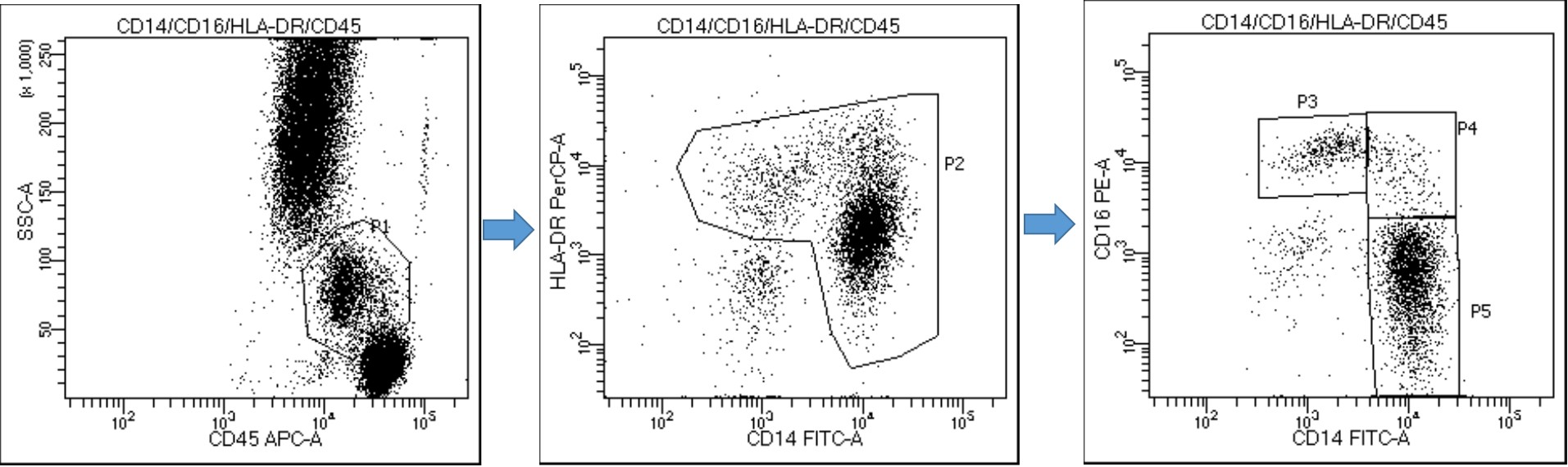

Supplement: Supplementary Figure 1 — FACS gating strategy for isolation of classical, intermediate and non-classical monocyte subsets. P1 – gate containing monocytes and NK cells; P2 – gate containing monocytes; P3,P4, P5 – gates containing non-classical, intermediate and classical monocytes respectively. [file Image_1.jpeg]

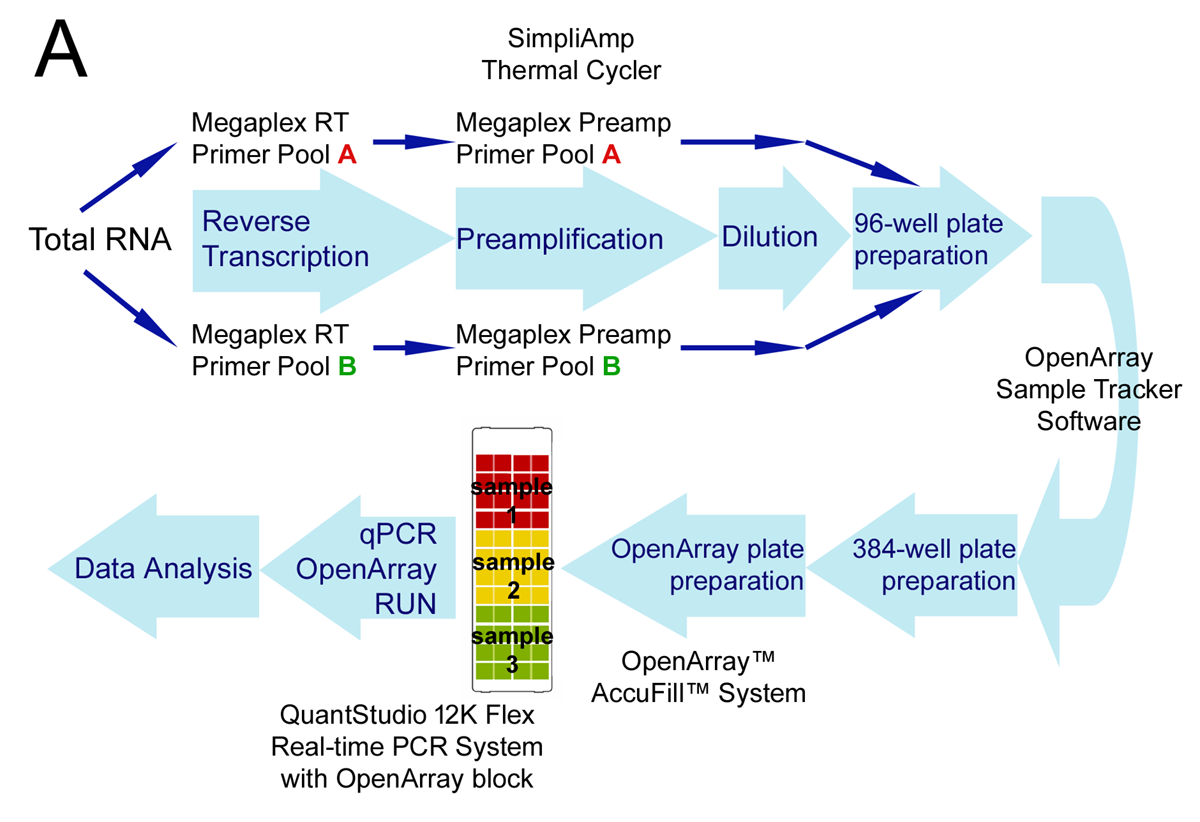

Supplement: Supplementary Figure 2 — OpenArray MicroRNA Expression Workflow presenting methodological aspects of miRNA expression analysis in human monocyte subpopulations. [file Image_2.tif]
