# Supplementary material for: Monocyte subpopulations display disease-specific miRNA signatures depending on the subform of Spondyloarthropathy
Source: Front Immunol. 2023 Apr 17;14:1124894. doi: 10.3389/fimmu.2023.1124894 (PMC10149963; doi:10.3389/fimmu.2023.1124894)
Supplement: Supplementary File 3 — Full list of miRNAs examined in this study. [file Presentation_1.pdf]

Unsupervised Principal Component Analysis (PCA) was performed in search for miRNA expression patterns that could be characteristic for SpA categories and/or Healthy individuals. PCA was run both with all differentially expressed miRNAs (regardless of the additional criteria described in the methodology section) as well as including differentially expressed miRNAs that were depicted in the manuscript's figure 1. The results of these analyses are shown below.

A. Considering all differentially expressed miRNAs:

PCA was performed and 3 major PC were extracted:

Classical monocytes

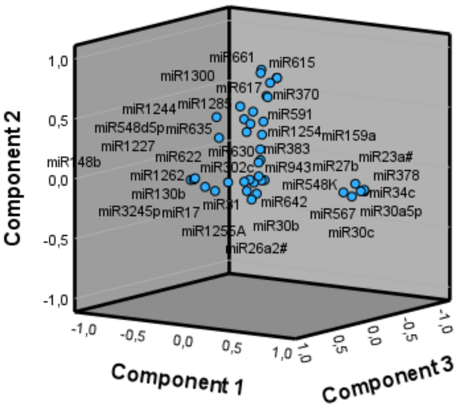

Intermediate monocytes

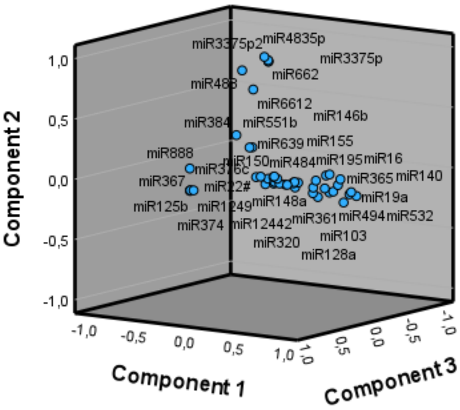

Non-classical monocytes

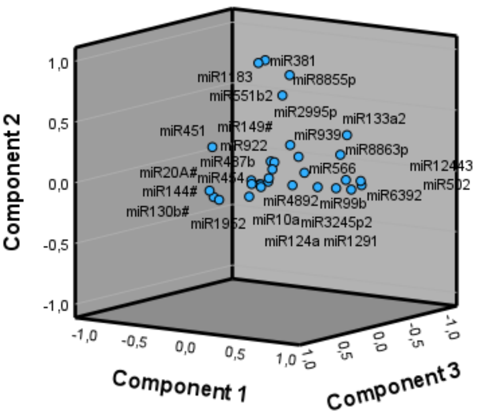

The clustering of some miRNAs within 3-dimensional space suggested the existence of similarities in miRNA expression patterns.
Next, the PC sores were extracted and analyzed for all the major PCs. PC scores were plotted to see whether there are any clear distribution patterns among AxSpA, perSpA and HC samples:

Classical monocytes

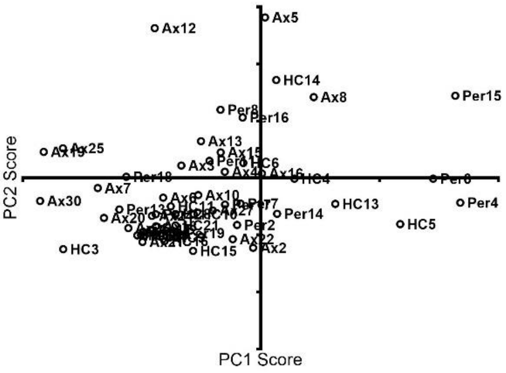

Intermediate monocytes

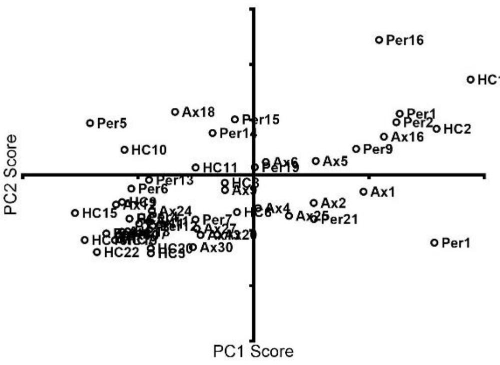

Non-classical monocytes

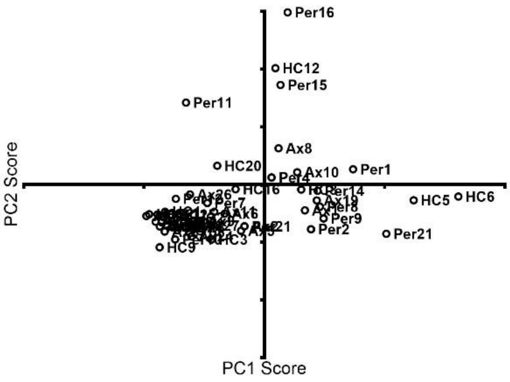

Although no clear grouping for AxSpA, perSpA and HC was observed, PC scores were further processed using multivariate analysis. Below, the results of multivariate analysis are presented (only the statistically significant results are shown). For this analysis samples were assigned as follows: perSpA = 1, AxSpA = 2, HC = 0.

For classical monocytes:

Multiple Comparisons								
				Mean Difference (I-J)	*Std. Error*	*Sig.*	*95% Confidence Interval*	
Dependent Variable		*(I)SpA*	*(J)SpA*				*Lower Bound*	*Upper Bound*
PC3 Score	*LSD*	*0*	*1*	*-.67304**	*0.319531*	*0.039*	*-1.31242*	*-0.03366*
			2	*-0.07008*	*0.300298*	*0.816*	*-0.67097*	*0.53082*
		1	*0*	*.67304**	*0.319531*	*0.039*	*0.03366*	*1.31242*
			2	*.60297**	*0.295668*	*0.046*	*0.01134*	*1.19460*
		2	*0*	*0.07008*	*0.300298*	*0.816*	*-0.53082*	*0.67097*
			1	*-.60297**	*0.295668*	*0.046*	*-1.19460*	*-0.01134*

No significant results for intermediate and non-classical monocytes were acquired.

As shown above expression patterns of miRNAs correlating the most with the PC3 in classical monocyte subset could potentially discriminate between perSpA vs axSpA and HC.

Based on the component loading values for PC3 (table below), these miRNAs would be:

miR-148b, miR-324-5p, miR-130b, miR-17, miR-30b, miR-1255, miR-302c, miR-26a-3p, miR-548a-5p

Rotated Component Matrix[a]			
	Component		
	1	*2*	*3*
miR-23a	*0.989*	*0.020*	*0.075*
miR 34c	*0.994*	*-0.060*	*-0.046*
miR 591	*0.037*	*0.446*	*0.225*
miR 567	*0.962*	*-0.063*	*-0.056*
miR 661	*-0.053*	*0.847*	*-0.063*
miR 615	*0.049*	*0.777*	*-0.143*
miR 630	*-0.002*	*0.201*	*0.027*
miR 943	*-0.026*	*-0.073*	*-0.070*
miR 617	*-0.050*	*0.586*	*0.246*
miR 30b	*0.432*	*-0.067*	*0.771*
miR 31	*-0.076*	*-0.142*	*0.114*
miR 27b	*-0.028*	*-0.029*	*0.011*
miR 1262	*-0.032*	*-0.046*	*0.213*
miR 302c	*-0.022*	*-0.013*	*0.461*
miR 17	*0.040*	*0.015*	*0.880*
miR 159a	*-0.025*	*0.312*	*-0.032*

miR 635	*-0.076*	*0.463*	*0.151*
miR 548d-5p	*-0.127*	*0.344*	*0.442*
miR 1300	*-0.024*	*0.830*	*-0.011*
miR 1227	*-0.075*	*0.351*	*0.118*
miR 383	*-0.042*	*0.098*	*-0.032*
miR 30c	*0.983*	*-0.081*	*0.116*
miR 370	*-0.028*	*0.612*	*-0.117*
miR 1285	*-0.101*	*0.500*	*-0.010*
miR 147b	*0.043*	*0.776*	*-0.146*
miR 205	*0.047*	*0.750*	*-0.045*
miR 642	*-0.058*	*-0.081*	*0.038*
miR 299-3p	*-0.035*	*-0.072*	*-0.039*
miR 1254	*-0.079*	*0.402*	*-0.129*
miR 26a-2-3p	*0.264*	*-0.080*	*0.457*
miR 548K	*0.878*	*-0.061*	*0.086*
miR 30a-5p	*0.994*	*-0.051*	*0.003*
miR 1255A	*0.020*	*-0.046*	*0.708*
miR 378	*0.994*	*-0.060*	*-0.046*
miR 190b	*-0.007*	*0.093*	*0.044*
miR 148b	*-0.016*	*0.091*	*0.945*
miR 1244	*-0.091*	*0.533*	*0.529*
miR 324-5p	*-0.029*	*0.078*	*0.944*
miR 130b	*-0.077*	*0.067*	*0.921*
miR 548a	*0.994*	*-0.057*	*-0.047*
miR 659	*-0.053*	*0.618*	*-0.135*
miR 638	*0.994*	*-0.048*	*-0.048*
miR 622	*-0.037*	*-0.041*	*0.130*
miR 548c	*0.994*	*-0.057*	*-0.046*
Extraction Method: Principal Component Analysis. Rotation Method: Varimax with Kaiser Normalization.[a]			
a. Rotation converged in 5 iterations.			

Performing the hierarchical clustering of miRNA expression data one might realize that expression profiles of 8 out of 9 depicted miRNAs in classical monocyte subset are closely related (picture below)

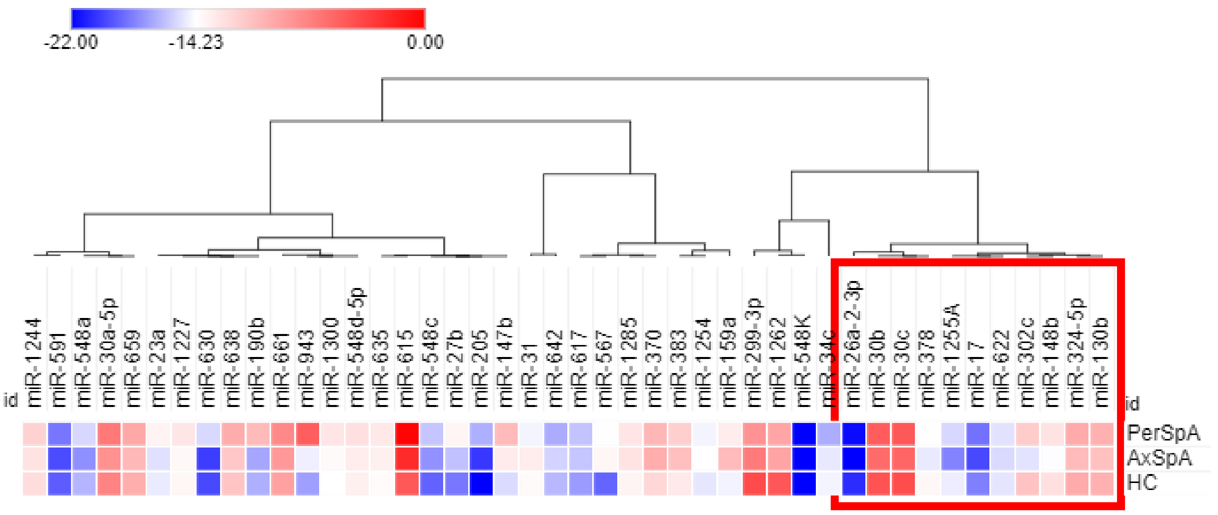

Conclusion: combined expression patterns of miR-148b, miR-324-5p, miR-130b, miR-17, miR-30b, miR-1255, miR-302c, miR-26a-3p in classical monocytes could potentially discriminate between perSpA vs. axSpA and HC

B. ***Including differentially expressed miRNAs fulfilling inclusion criteria described in the M&M section of the manuscript:***

The analysis was performed as described above. PCA was performed and 3 major PC were extracted:

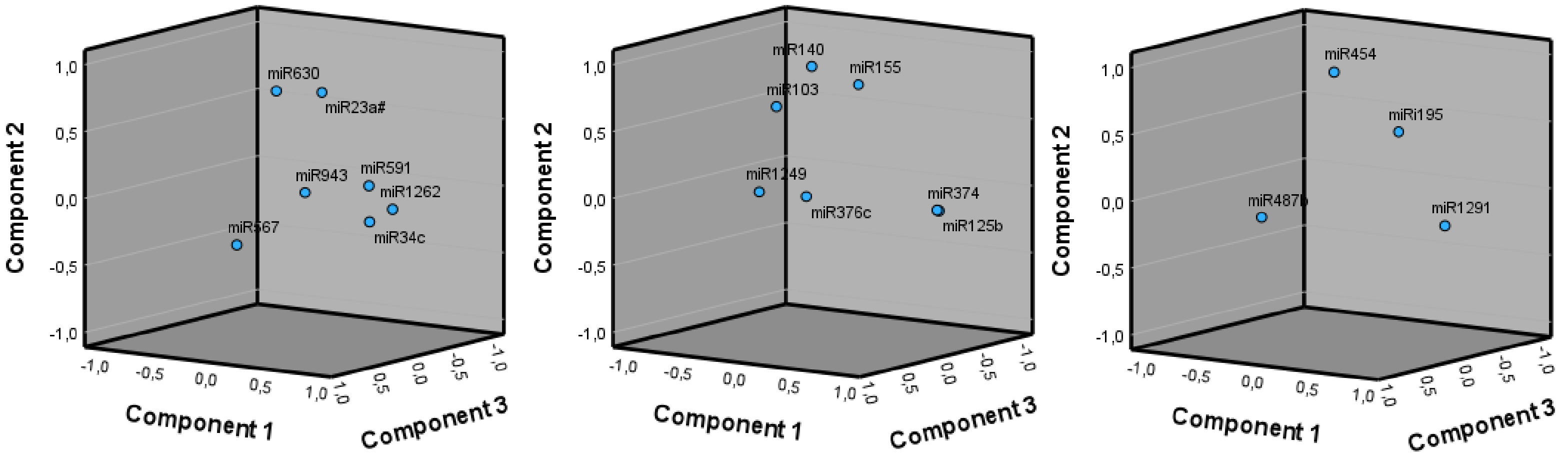

Next, the PC sores were extracted and analyzed for all the major PCs. PC scores were plotted to see whether there are any clear distribution patterns among AxSpA, perSpA and HC samples:

Classical monocytes

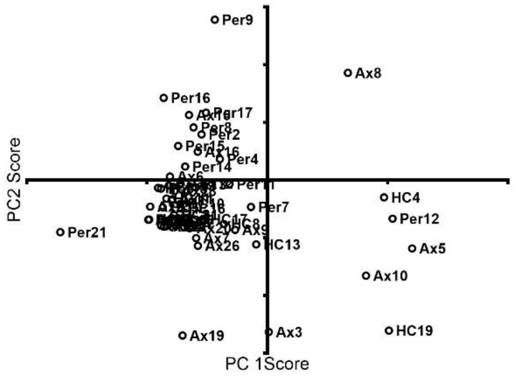

Intermediate monocytes

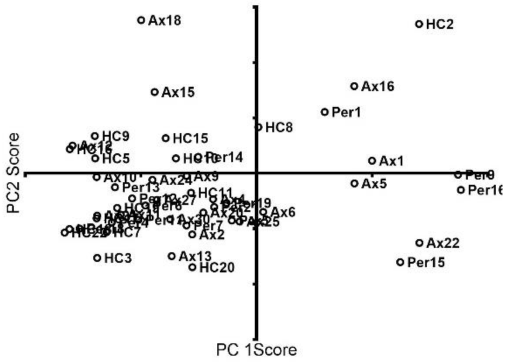

Non-classical monocytes

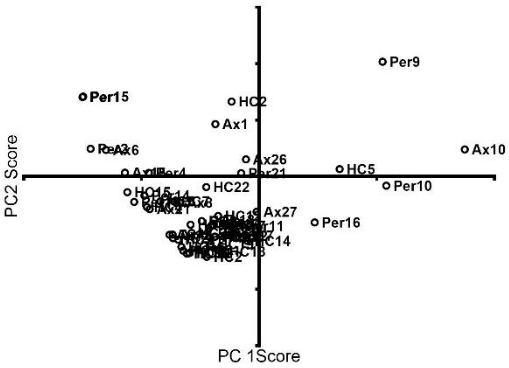

Although no clear grouping for AxSpA, perSpA and HC was observed, PC scores were further processed using multivariate analysis. Below, the results of multivariate analysis are presented (only the statistically significant results are shown). For this analysis samples were assigned as follows: perSpA = 1, AxSpA = 2, HC = 0.

For classical monocytes:

**Multiple Comparisons**								
				Mean	Std.	Sig.	95% Confidence Interval	
Dependent Variable		(I)SpA	(J)SpA	Difference (I-J)	Error		Lower Bound	Upper Bound
PC2 Score	LSD	0	1	-.85610*	0.303223	0.006	-1.46285	-0.24936
			2	0.08356	0.284972	0.770	-0.48666	0.65379
		1	0	.85610*	0.303223	0.006	0.24936	1.46285
			2	.93967*	0.280578	0.001	0.37823	1.50110
		2	0	-0.08356	0.284972	0.770	-0.65379	0.48666
			1	-.93967*	0.280578	0.001	-1.50110	-0.37823

For Intermediate monocytes:

				Mean Difference (I-J)	Std. Error	Sig.	95% Confidence Interval	
Dependent Variable		(I)SpA	(J)SpA				Lower Bound	Upper Bound
PC3 Score	LSD	0	1	-.81212*	0.3143	0.0124	-1.4418	-0.1825
			2	-.63031*	0.2993	0.0397	-1.2299	-0.0308
		1	0	.81212*	0.3143	0.0124	0.1825	1.4418
			2	0.1818	0.3037	0.5518	-0.4266	0.7902
		2	0	.63031*	0.2993	0.0397	0.0308	1.2299
			1	-0.1818	0.3037	0.5518	-0.7902	0.4266

For Non-Classical monocytes:

				Mean Difference (I-J)	Std. Error	Sig.	95% Confidence Interval	
Dependent Variable		(I)SpA	(J)SpA				Lower Bound	Upper Bound
PC1 Score	LSD	0	1	-0.0202	0.3273	0.9510	-0.6771	0.6367
			2	-,79861*	0.2935	0.0088	-1.3876	-0.2096
		1	0	0.0202	0.3273	0.9510	-0.6367	0.6771
			2	-,77842*	0.3241	0.0199	-1.4288	-0.1280
		2	0	,79861*	0.2935	0.0088	0.2096	1.3876
			1	,77842*	0.3241	0.0199	0.1280	1.4288
PC3 Score	LSD	0	1	-0.2796	0.3350	0.4078	-0.9519	0.3926
			2	-,75370*	0.3004	0.0153	-1.3565	-0.1509
		1	0	0.2796	0.3350	0.4078	-0.3926	0.9519
			2	-0.4741	0.3317	0.1589	-1.1397	0.1915
		2	0	,75370*	0.3004	0.0153	0.1509	1.3565
			1	0.4741	0.3317	0.1589	-0.1915	1.1397

As shown above:

- *For Classical monocytes expression patterns of miRNAs correlating the most with the PC2 could potentially discriminate between perSpA vs axSpA and HC.*

- *For Intermediate monocytes expression patterns of miRNAs correlating the most with the PC3 could potentially discriminate between HC vs axSpA and perSpA.*

- *For non-Classical monocytes expression patterns of miRNAs correlating the most with the PC1 could potentially discriminate between axSpA vs perSpA and HC. Also PC3 could discriminate between axSpA vs HC.*

Based on the component loading values for corresponding PCs these miRNAs would be:

***miR-23a and miR-630 –** for classical monocytes*

***miR-1249 –** for intermediate monocytes*

***miR-195 and miR-1291** for PC1 and **miR-487b** for PC3 – for non-classical monocytes*

*Performing the hierarchical clustering of miRNA expression data one might realize that expression profiles of **miR-23a and miR-630 in** classical monocyte subset are closely related (picture below). On the other hand in non-classical monocytes, expression profiles of **miR-195 and miR-1291 cluster together, whereas miR-487b** is separated from others miRNAs.*

Classical monocytes Intermediate monocytes Non-classical monocytes

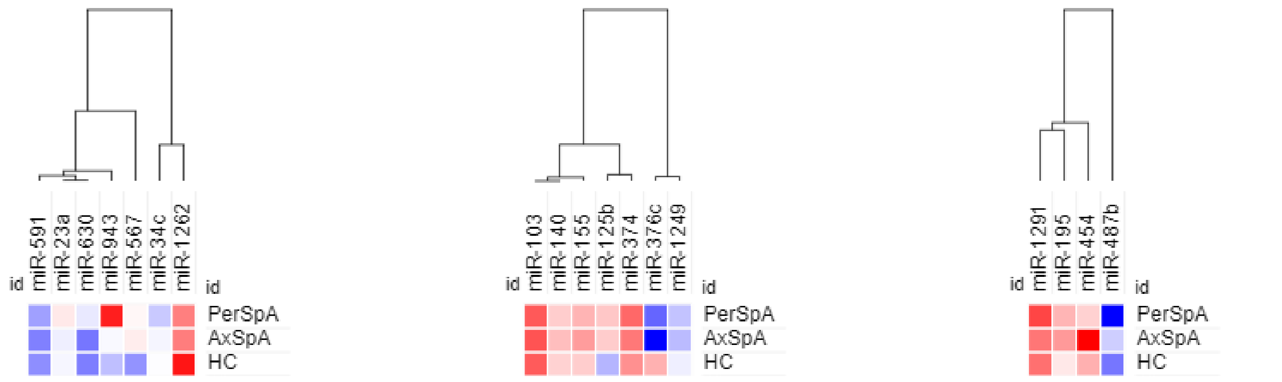

Concluding – based on Principal Component Analysis –

a) *expression patterns of **combined miR-23a and miR-630 in classical monocyte subset** could serve as a good discriminator between perSpA vs axSpA and HC.*
b) *Expression pattern of **miR-1249 in intermediate monocyte subset** could well discriminate between HC vs axSpA and perSpA.*
c) *Combined expression patterns of **miR-195 and miR-1291 in non-classical monocyte subset** could serve as a good discriminator between axSpA vs perSpA and HC. On the other hand differential expression of **miR-487b** could be used to discriminate between axSpA vs HC.*
